# Grand Challenges in Neurotechnology and System Neuroergonomics

**DOI:** 10.3389/fnrgo.2020.602504

**Published:** 2020-11-30

**Authors:** Stephen H. Fairclough, Fabien Lotte

**Affiliations:** ^1^Liverpool John Moores University, Liverpool, United Kingdom; ^2^Inria Bordeaux Sud-Ouest, Talence, France; ^3^LaBRI (CNRS/Univ. Bordeaux/Bordeaux INP), Bordeaux, France

**Keywords:** EEG, fNIRS, human-computer interaction, brain-computer interfaces, neurotechnologies, neuroergonomics

## 1. Introduction

The research field of Neuroergonomics aims at “*Understanding the brain in the wild, its activity during unrestricted real-world tasks in everyday life contexts, and its relationship to action, behavior, body, and environment*” (Dehais et al., 2020). This field has tremendous potential to develop innovative applications across many fields, such as education, manufacturing, entertainment, health, transportation. In order to achieve this potential, many research applications of Neuroergonomics rely on or require neurotechnologies. Neurotechnology is a category of technology where system design incorporates neural principles or directly interfaces with signals from the brain and body. The most popular types of neurotechnologies notably include Brain-Computer Interfaces (BCI) (Clerc et al., 2016a,b; Nam et al., 2018) and Physiological computing (Fairclough, 2009; Fairclough and Gilleade, 2014). In order to be used in practice, Neuroergonomics must also be studied and integrated at the whole system level. In other words, we need to develop concepts of Systems Neuroergonomics, an interdisciplinary field of engineering, neuroscience and human factors, which integrates approaches from Neuroergonomics into the design, development, and management of complex systems (e.g., planes, information systems, video games, or medical devices). Systems in this context refers to any combination of machines, robots, computers, and automation with human users. The present journal, Frontiers in Neuroergonomics, section Neurotechonology and System Neuroergonomics, aims to publish significant advances in those principles, protocols, and applications that underpin the development of neurotechnology in the context of Neuroergonomics, i.e., to create novel forms of human-computer interaction that could enhance, e.g., safety, productivity, or health.

**Figure 1:**
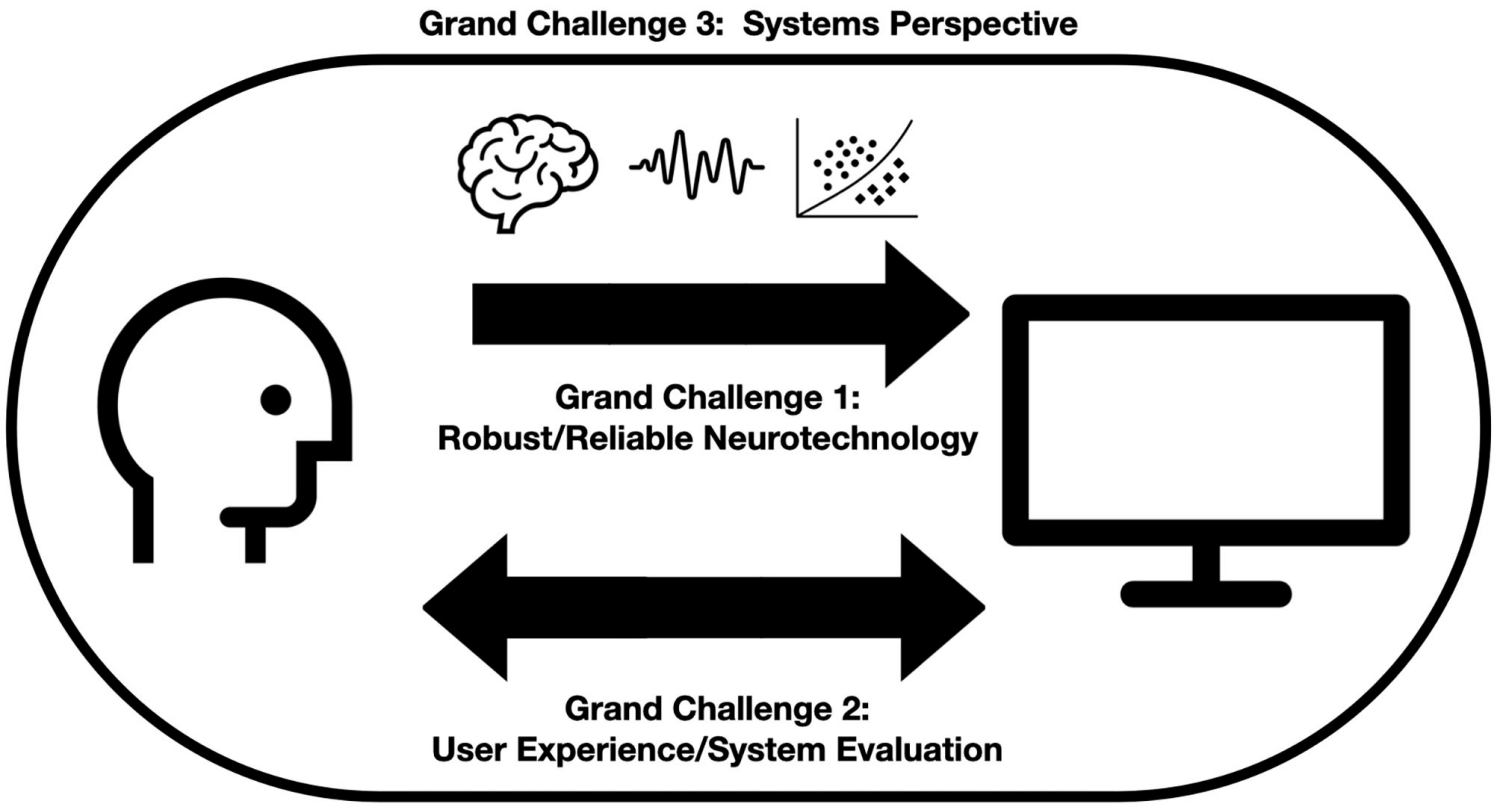
A graphic illustration of the three grand challenges (GC) for Neurotechnology and Systems Neuroergonomics.

However, this objective remains far from the reality of how technologies are integrated into work and leisure in everyday life. Indeed, the vast majority of neurotechnologies for Neuroergonomics remain at the level of demonstrator systems used for laboratory research, and very few are used outside those laboratories. If Neuroergonomics, by definition, aims at studying behavior and technologies as they are used *in the wild*, it is important for neurotechnologies to also make this developmental leap from the laboratory and into the real world. Moreover, we must develop a systems understanding of how neurotechnologies may be embedded in the work of the individual, the team, and the organization. In order to understand how neurotechnologies can evolve from their current status as laboratory demonstrators to usage cases in everyday work and leisure, we have proposed three grand challenges: (1) Designing neurotechnologies that are robust and reliable, even outside the lab, with high accuracy across all usage contexts. This is a challenge at the machine level. (2) Designing user experience with neurotechnologies, to ensure that these technologies are usable, acceptable, and useful for its users. This is a challenge at the user level. (3) Developing Systems thinking in Neuroergonomics, to integrate, study, and optimize Neuroergonomics into system design, in a principled way. This is a challenge at the overall system level. These grand challenges are illustrated in Figure 1 and are described in more details in the following sections.

## 2. Grand Challenge: Designing Robust and Reliable Neurotechnologies

Despite their potential, current neurotechnologies are still far from being sufficiently reliable for use in everyday work and life. Existing systems make frequent and numerous mistakes in decoding the users' mental states or intentions from their neurophysiological signals (Allanson and Fairclough, 2004; Krusienski et al., 2011; Lotte, 2016; Chavarriaga et al., 2017; Lotte et al., 2018). This poor reliability is due to a number of factors, such as imperfect neurophysiological sensors, notably Electroencephalography (EEG) and functional Near Infrared Spectroscopy (fNIRS) which only record relatively low Signal-to-Noise-Ratio (SNR) data. These types of physiological signals are very sensitive to various types of systemic artifacts originating from non-neuronal sources, e.g., muscles (Electromyography—EMG), eyes (Electrooculography—EOG), or motions for EEG (Goncharova et al., 2003; Fatourechi et al., 2007) or from various light sources or motions for fNIRS (Vitorio et al., 2017; Hocke et al., 2018), or from the environment itself (Sweeney et al., 2012). This deterioration of neurophysiological signals by artifacts is even more pronounced in real-life situations, with potentially mobile users and noisy environments (Lotte et al., 2009; Strait and Scheutz, 2014; Minguillon et al., 2017), which also represent the target use case of Neuroergonomics. While various approaches have been proposed to deal with artifacts in the neurophysiological record (see e.g., Cooper et al., 2012; Sweeney et al., 2012; Urigüen and Garcia-Zapirain, 2015; Jiang et al., 2019), these corrective strategies are still far from being perfect and do not remove the whole artifact, i.e., a residual artifact remains, or they risk accidentally removing a relevant part of the signal. Finally, neurophysiological signals are also highly non-stationary with significant variation between different users and within the same user, e.g., between days or even within the same day (see Abu-Alqumsan et al., 2017; Krumpe et al., 2017; Mladenovic et al., 2018; Saha and Baumert, 2019).

The goal of this grand challenge is to design robust neurotechnology that can decode users' states reliably during everyday work and leisure, despite those various sources of noise, artifacts, and non-stationarity that affect the recorded signals. This goal can be decomposed into a number of sub-challenges. The first sub-challenge is to design new sensors that can record neurophysiological signals with higher SNR. In addition, in order to be able to deal with the various sources of noise, artifacts, and non-stationarity, we must be able to build detailed computational models in order to characterize them. What are their spatial, temporal or spectral characteristics? What are their causes? What are their specific influences on neurophysiological signals? Current research has provided partial answers to some of these questions (see, e.g., Grosse-Wentrup, 2011; Ahn and Jun, 2015; Jeunet et al., 2017), but in order to design robust neurotechnologies in an informed way, we require computational models that are detailed and predictive. These types of models would enable us to optimize the design of algorithms to detect noise sources and artifacts for specific signals in a particular context (e.g., task, environment), and allow us to remove them in order to yield “clean” neurophysiological signals. The same knowledge could also be used to design machine learning and signal processing algorithms that are robust to specific sources of artifacts and noise, or even to extract neurophysiological features that are invariant to these noise sources, extending those ideas initiated by Blankertz et al. (2008) and Lotte and Guan (2011). Similarly, models of non-stationarities would enable us to track them more finely, and in doing so, design decoding algorithms that are not strongly influenced by their presence. Alternatively, we could design adaptive machine learning algorithms (Shenoy et al., 2006; Lotte et al., 2018), that would update parameters in an informed way in order to accommodate non-stationarities in their source signals. It is important to note that we would require such algorithms for both off-line data (for off-line evaluations and/or more fundamental neuroscientific studies) but also for their online equivalents, for integration into real-time neurotechnologies. Therefore, another sub-challenge that relates specifically to the online case is the requirement to design algorithms that are sufficiently computationally efficient to be used online.

The design of reliable and robust neurotechnologies also requires the design of algorithms that can exploit available data in an optimal fashion. For example, the design of algorithms based on multimodal data that jointly exploit multiple types of neurophysiological signals within the same process of classification, e.g., combining features from EEG, fNIRS, electrocardiogram (ECG), or galvanic skin response (GSR), in order to amalgamate the particular strengths of each. In other words, we need to design versatile and effective Hybrid BCIs (Pfurtscheller et al., 2010; Banville and Falk, 2016). We also need to design algorithms that can be calibrated or trained with as little data as possible, given how little data is typically available prior to sustained usage by an individual. A classification algorithm that could be efficiently trained (i.e., from little data) would also reduce the calibration times of current neurotechnologies (Lotte, 2015). Ideally, the aim for neurotechnologies that are used in everyday life should be removing altogether the need for new data for each new user, using, e.g., transfer learning (Jayaram et al., 2016). Since neurotechnologies for neuroergonomics often aim at decoding specific mental states, we would need to identify biomarkers that are specific to those mental states, but do not vary with co-existing states, thus ensuring their robust decoding (Fairclough, 2009). Finally, in order to be able to design and build all the models and algorithms described in this section, we would need large databases that represent neurophysiological data collected in the field. Therefore, the final sub-challenge would be to collect, build, consolidate, and share large databases of neurophysiological and psychophysiological signals, which have been measured across many users, tasks, contexts, and real operational environments (e.g., in planes or cars).

## 3. Grand Challenge: Designing User Experience With Neurotechnologies

Issues associated with the user experience represents a significant challenge for the development of neurotechnologies. This is an important issue because all nascent technologies must offer tangible benefits in order to achieve widespread adoption, and these benefits are manifested via the user experience. The first part of this challenge concerns the design of those peripherals used for neurotechnology. Wearable sensors must be designed for comfort, robustness, aesthetic design, and unobtrusive measurement that does not compromise the quality of data collection (Mihajlović et al., 2015). In practice, this balance can be difficult to achieve, especially when sensors must be used over long periods of time in everyday/public environments. As a secondary point, these wearable devices, which are designed to exchange data with other devices, must encompass reliable communication protocols that are easily installed by members of the general public.

While neurotechnology has enormous potential to enhance human-computer interaction (HCI) by expanding communication bandwidth and developing personalized modes of interaction, acceptance depends largely on which adaptive strategies are deployed (Fuchs, 2018) and users' perceptions of their utility and effectiveness. At the present time, we know relatively little about the level of system error that is acceptable to users when they interact with neurotechnology (Évain et al., 2016). When errors do occur, error recovery mechanisms must be seamlessly integrated into the interaction with the user. Similarly, we lack a strong understanding of how classification rates derived from machine learning algorithms regarding the internal state of the user correspond with those subjective types of self-assessment that informs perceived accuracy of the system (Fairclough et al., 2015; McCrea et al., 2016). Due to the high speed of data exchange between brain and machine, interactions with neurotechnology can occur implicitly and autonomously, i.e., functions can be activated without seeking confirmation from the user (Solovey et al., 2015; Serim and Jacucci, 2019). While this is a potentially exciting development from a HCI perspective, we know relatively little about how users will respond to this type of interaction mechanic, will they welcome an opportunity to communicate unconsciously with a machine or experience the triggering of autonomous functions by real-time changes in neurophysiological activity as a loss of personal control?

Research into interface design principles for neurotechnologies remains largely unexplored with the exception of active control BCI (Mason and Birch, 2003; Zander and Kothe, 2011). The provision of feedback to the user is a particularly important component of interface design. Feedback allows users to understand the internal contingencies of the system (Pillette, 2019), but given the high-throughput of data during these interactions, how can feedback be delivered at the interface without overwhelming the individual with information? From the perspective of the user, working with a neurotechnology is a learning experience that unfolds over a sustained period of use; but at the time of writing, we have very little understanding of how this longitudinal dimension influences user behavior. If users receive neurophysiological feedback over a sustained period of repeated interactions, it is possible for them to learn how to self-regulate brain activity in order to achieve a desired outcome, as would be the case with conventional neurofeedback; however, we are currently unable to assess the viability of self-regulation as an interaction mechanic beyond the mental and motor imagery protocols, which characterize active BCI (Cavazza et al., 2014). Similarly, sustained exposure to a working neurotechnology permits the user to assess an appropriate degree of trust in the system, which is likely to be highly significant for those systems associated with autonomous function (Lee and See, 2004; Hoff and Bashir, 2015) and can be assessed using psychophysiological (Hu et al., 2016) and neurophysiological (de Visser et al., 2018) measures.

The evaluation of working neurotechnology presents another set of challenges for the researcher. The whole purpose of this technology is to enhance or extend existing modalities of human-machine communication. In order to demonstrate that HCI have been improved or enhanced, neurotechnology must be evaluated, but this type of assessments is relative and must be benchmarked to an appropriate point of comparison. The development of methodologies and measures to permit the evaluation of neurotechnology remains mostly unexplored (Chavarriaga et al., 2017). There is a past precedent for using randomized inputs or “yoked” controls for the purposes of comparison (Scerbo et al., 2003; Zander et al., 2016) but these methods await consolidation within a generic framework for the evaluation of neurotechnology. Metrics for evaluation remain at a similar level of immaturity and these metrics can be complex for systems that blur the traditional distinction between user and technology (Fairclough, 2015). A working neurotechnology represents a hybrid system where human and machine operate as a single “cooperative intelligent entity” (p. 96) (Hancock, 2009). The generative interplay between “live” neurophysiological data and the adaptive logic of technology lies at the crux of user interaction and is quantified by observing the behavior of the system; hence the behavior of the adaptive system itself represents a central metric for system evaluation (Ewing et al., 2016).

## 4. Grand Challenge: Systems Thinking in Neuroergonomics

A systems perspective on the development of neurotechnology can be understood at two levels of analyses. In the first instance, neurotechnology work on a closed-loop basis wherein data is collected from the user, transformed and relayed to the individual via events at the interface. This type of biocybernetic system (Pope et al., 1995) is characterized as a control loop where the boundary between person and machine is rendered porous to enable a symmetrical mode of human-computer interaction (Hettinger et al., 2003). This bidirectional exchange of data can be extended by incorporating concepts from neural function and neural architecture into the technical specification and design of the system. For example, deep learning is a machine learning method designed to emulate representational learning in a way that parallels computation in the brain; this method has been incorporated into the design of BCI (Roy et al., 2019; Zhang et al., 2019) and there may be other features of neural computation that will transfer to the design of neurotechnology.

The second challenge involves broadening the context of system usage to consider neurotechnology as a sociotechnical system. If neurotechnology is to be widely adopted, it is important to anticipate how it may function for different users operating across multiple contexts (e.g., work vs. leisure) and being subject to those legal/governance issues that operate in the real-world. At the current time, research on neurotechnology tends to ignore this broad context, although there are exceptions (e.g., IEEE, 2020). Neurotechnology must operate within a sociotechnical hierarchy that includes protocols and procedures defined by managements, companies, regulators, and government (Rasmussen, 1997). The influence of this hierarchy is impossible to reproduce in the laboratory and sociotechnical issues must be studied “in the wild” using real employees incorporating neurotechnology into existing work practices (Wilson, 2014). Understanding the influence of the sociotechnical hierarchy on the design, functionality and usage of neurotechnology is an important challenge for the field in general and the development of commercial systems in particular.

By expanding our conception of how neurotechnology works to include real-world constraints, we are striving to “future-proof” system concepts and working prototypes. Interactions between neurotechnologies and other components, which may be social, organizational, economic, or political in nature, can only be observed in the real world. In addition, the closed-loop character of neurotechnology already occupies the boundary between a complicated system (i.e., a system that is difficult to understand but can be decomposed) and a complex one (i.e., a system that can evolve behavior and behave in ways unanticipated by the designer). The “darkness principle” (Siemieniuch and Sinclair, 2006) states that “no system can be known completely” and this principle is pertinent to the adoption and utilization of neurotechnology in the field across various settings and tasks. In addition to patterns of unanticipated usage that are often observed under real-world constraints of time, space, protocols, and procedures (Wilson, 2014), there are various ways in which the technical properties of the system can influence procedures in a bottom-up direction at the higher levels of sociotechnical hierarchy, e.g., requirement for new processes or laws. This complexity requires the researcher to maintain a holistic perspective, understanding how neurotechnology influences health, duration of working hours, liability for error etc. as well as the traditional emphasis on performance efficiency. This perspective also represents a tacit argument for a participatory design approach wherein prospective users have an opportunity to experience the system and provide feedback on how neurotechnology should be designed and integrated into work or lifestyle practices.

## 5. Summary and Conclusions

In this article, we have considered the challenges of developing robust categories of neurotechnology that can be utilized in the real-world. These systems range from active BCI to passive forms of neuroadaptive technologies, and encompass related system that incorporate measurement of peripheral physiology, such as physiological computing and affective computing. These systems are united by the need for wearable sensors, a requirement to assess the cognitive/emotional state of the user in real-time and a bidirectional flow of information between user and system that can be described as a closed-loop. If neuroergonomics represents the study of the brain in the context of everyday life, neurotechnology extends this approach by turning those lessons learned from that field of study into novel forms of communication between user and system. However, there are numerous challenges facing the successful transition of this nascent technology from the laboratory into working systems that are utilized by people on an everyday basis. Methods for measuring neurophysiology and psychophysiology were developed originally for use under controlled conditions, the first grand challenge for neurotechnology is the creation of sensors combined with mathematical methods that can deliver robust data in the real-world. If this challenge was achieved and we could obtain accurate neurophysiological data from everyday life, the next problem involves a conversion of that technical achievement into novel modes of human-computer interaction that offer genuine utility for the user. In other words, neurotechnology should enhance performance or health or enjoyment in a way that is genuinely beneficial and cannot be achieved by some other means. The benefits of using neurotechnology must be both tangible and unique. In order to explore how neurotechnology can create positive user experiences, we must develop our understanding of longitudinal use, the development of trust and the design of appropriate methods to enable system evaluation. Once we have a robust neurotechnology that seems to deliver real utility for a specific user or groups of user, it is important to consider the sociotechnical context of system use with respect to organizational, economic, and political structures. Standards of governance for neurotechnologies can be informed by recommendations for ethical AI (DIB, 2019). Neurotechnology requires privileged access to data sources that are covert, rich and sensitive to a variety of influences and it is important that standards of governance and data privacy keep pace with practices if this emergent technology is adopted at scale.

## Author Contributions

All authors contributed equally to the intellectual efforts and writing of the paper.

## Conflict of Interest

The authors declare that the research was conducted in the absence of any commercial or financial relationships that could be construed as a potential conflict of interest.

## References

[B1] Abu-Alqumsan M. Kapeller C. Hintermüller C. Guger C. Peer A. (2017). Invariance and variability in interaction error-related potentials and their consequences for classification. J. Neural Eng. 14:066015. 10.1088/1741-2552/aa8416 28776500

[B2] Ahn M. Jun S. C. (2015). Performance variation in motor imagery brain-computer interface: a brief review. J. Neurosci. Methods 243, 103–110. 10.1016/j.jneumeth.2015.01.033 25668430

[B3] Allanson J. Fairclough S. H. (2004). A research agenda for physiological computing. Interact. Comput. 16, 857–878. 10.1016/j.intcom.2004.08.001

[B4] Banville H. Falk T. (2016). Recent advances and open challenges in hybrid brain-computer interfacing: a technological review of non-invasive human research. Brain-Comput. Interfaces, 3, 9–46. 10.1080/2326263X.2015.1134958

[B5] Blankertz B. Kawanabe M. Tomioka R. Hohlefeld F. Müller K.-R. Nikulin V. V. (2008). “Invariant common spatial patterns: alleviating nonstationarities in brain-computer interfacing,” in Advances in Neural Information Processing Systems (Vancouver, BC), 113–120.

[B6] Cavazza M. Charles F. Gilroy S. W. Porteous J. Aranyi G. Raz G.. (2014). “Integrating virtual agents in BCI neurofeedback systems,” in Proceedings of the 2014 Virtual Reality International Conference, VRIC'14 (New York, NY: Association for Computing Machinery). 10.1145/2617841.2620713

[B7] Chavarriaga R. Fried-Oken M. Kleih S. Lotte F. Scherer R. (2017). Heading for new shores! overcoming pitfalls in BCI design. Brain Comput. Interfaces 1–14. 10.1080/2326263X.2016.1263916 29629393 PMC5884128

[B8] Clerc M. Bougrain L. Lotte F. (2016a). Brain-Computer Interfaces 1: Foundations and Methods (London:ISTE-Wiley). 10.1002/9781119144977

[B9] Clerc M. Bougrain L. Lotte F. (2016b). Brain-Computer Interfaces 2: Technology and Applications (Hoboken, NJ:ISTE-Wiley). 10.1002/9781119332428

[B10] Cooper R. Selb J. Gagnon L. Phillip D. Schytz H. W. Iversen H. K.. (2012). A systematic comparison of motion artifact correction techniques for functional near-infrared spectroscopy. Front. Neurosci. 6:147. 10.3389/fnins.2012.00147 23087603 PMC3468891

[B11] de Visser E. J. Beatty P. J. Estepp J. R. Kohn S. Abubshait A. Fedota J. R.. (2018). Learning from the slips of others: neural correlates of trust in automated agents. Front. Hum. Neurosci. 12:309. 10.3389/fnhum.2018.00309 30147648 PMC6095965

[B12] Dehais F. Karwowski W. Ayaz H. (2020). Brain in the wild as the next frontier: grand field challenges for neuroergonomics. Front. Neuroergon. 1:583733. 10.3389/fnrgo.2020.583733 PMC10790928 38234310

[B13] DIB (2019). AI Principles: Recommendations on the Ethical Use of Artificial Intelligence by the Department of Defense. Technical report, DIB.

[B14] Évain A. Argelaguet F. Strock A. Roussel N. Casiez G. Lécuyer A. (2016). “Influence of error rate on frustration of BCI users,” in Proceedings of the International Working Conference on Advanced Visual Interfaces, AVI'16 (New York, NY: Association for Computing Machinery), 248–251. 10.1145/2909132.2909278

[B15] Ewing K. C. Fairclough S. H. Gilleade K. (2016). Evaluation of an adaptive game that uses EEG measures validated during the design process as inputs to a biocybernetic loop. Front. Hum. Neurosci. 10:223. 10.3389/fnhum.2016.00223 27242486 PMC4870503

[B16] Fairclough S. (2015). “A closed-loop perspective on symbiotic human-computer interaction,” in Symbiotic Interaction, eds B. Blankertz, G. Jacucci, L. Gamberini, A. Spagnolli, and J. Freeman (Cham: Springer International Publishing), 57–67. 10.1007/978-3-319-24917-9_6

[B17] Fairclough S. H. (2009). Fundamentals of physiological computing. Interact. Comput. 21, 133–145. 10.1016/j.intcom.2008.10.011

[B18] Fairclough S. H. Gilleade K. (2014). Advances in Physiological Computing (London: Springer). 10.1007/978-1-4471-6392-3

[B19] Fairclough S. H. Karran A. J. Gilleade K. (2015). “Classification accuracy from the perspective of the user: real-time interaction with physiological computing,” in Proceedings of the 33rd Annual ACM Conference on Human Factors in Computing Systems, CHI'15 (New York, NY: Association for Computing Machinery), 3029–3038. 10.1145/2702123.2702454

[B20] Fatourechi M. Bashashati A. Ward R. Birch G. (2007). EMG and EOG artifacts in brain computer interface systems: a survey. Clin. Neurophysiol. 118, 480–494. 10.1016/j.clinph.2006.10.019 17169606

[B21] Fuchs S. (2018). “Session overview: adaptation strategies and adaptation management,” in Augmented Cognition: Intelligent Technologies, eds D. D. Schmorrow and C. M. Fidopiastis (Cham: Springer International Publishing), 3–8. 10.1007/978-3-319-91470-1_1

[B22] Goncharova I. McFarland D. Vaughan T. Wolpaw J. (2003). EMG contamination of EEG: spectral and topographical characteristics. Clin. Neurophysiol. 114, 1580–1593. 10.1016/S1388-2457(03)00093-2 12948787

[B23] Grosse-Wentrup M. (2011). What are the causes of performance variation in brain-computer interfacing? Int. J. Bioelectromagn. 13, 115–116.

[B24] Hancock P. A. (2009). Mind, Machine and Morality: Towards a Philosophy of Human-Technology Symbiosis (London: Ashgate).

[B25] Hettinger L. J. Branco P. Encarnaco L. M. Bonato P. (2003). Neuroadaptive technologies: applying neuroergonomics to the design of advanced interfaces. Theor. Issues Ergon. Sci. 4, 220–237. 10.1080/1463922021000020918

[B26] Hocke L. M. Oni I. K. Duszynski C. C. Corrigan A. V. Frederick B. d. Dunn J. F. (2018). Automated processing of fNIRS data-a visual guide to the pitfalls and consequences. Algorithms 11:67. 10.3390/a11050067 30906511 PMC6428450

[B27] Hoff K. A. Bashir M. (2015). Trust in automation: Integrating empirical evidence on factors that influence trust. Hum. Fact. 57, 407–434. 10.1177/0018720814547570 25875432

[B28] Hu W.-L. Akash K. Jain N. Reid T. (2016). Real-time sensing of trust in human-machine interactions. IFAC-PapersOnLine 49, 48–53. 10.1016/j.ifacol.2016.12.188

[B29] IEEE (2020). Standards Roadmap: Neurotechnologies for Brain-Machine Interfacing (New York, NY).

[B30] Jayaram V. Alamgir M. Altun Y. Scholkopf B. Grosse-Wentrup M. (2016). Transfer learning in brain-computer interfaces. IEEE Comput. Intell. Mag. 11, 20–31. 10.1109/MCI.2015.2501545

[B31] Jeunet C. N'Kaoua B. Lotte F. (2017). “Towards a cognitive model of MI-BCI user training,” in 7th International BCI Conference (Graz).

[B32] Jiang X. Bian G.-B. Tian Z. (2019). Removal of artifacts from EEG signals: a review. Sensors 19:987. 10.3390/s19050987 30813520 PMC6427454

[B33] Krumpe T. Baumgaertner K. Rosenstiel W. Spüler M. (2017). “Non-stationarity and inter-subject variability of EEG characteristics in the context of BCI development,” in Proc. Int Graz BCI Conference (Graz).

[B34] Krusienski D. Grosse-Wentrup M. Galán F. Coyle D. Miller K. Forney E.. (2011). Critical issues in state-of-the-art brain-computer interface signal processing. J. Neural Eng. 8:025002. 10.1088/1741-2560/8/2/025002 21436519 PMC3412170

[B35] Lee J. D. See K. A. (2004). Trust in automation: designing for appropriate reliance. Hum. Fact. 46, 50–80. 10.1518/hfes.46.1.50.30392 15151155

[B36] Lotte F. (2015). Signal processing approaches to minimize or suppress calibration time in oscillatory activity-based brain-computer interfaces. Proc. IEEE 103, 871–890. 10.1109/JPROC.2015.2404941

[B37] Lotte F. (2016). Towards usable electroencephalography-based brain-computer interfaces (Habilitation thesis). Habilitation á diriger des Recherches (HDR), University of Bordeaux, Bordeaux, France.

[B38] Lotte F. Bougrain L. Cichocki A. Clerc M. Congedo M. Rakotomamonjy A.. (2018). A review of classification algorithms for EEG-based brain-computer interfaces: a 10-year update. J. Neural Eng. (Athens), 15:031005. 10.1088/1741-2552/aab2f2 29488902

[B39] Lotte F. Fujisawa J. Touyama H. Ito R. Hirose M. Lécuyer A. (2009). “Towards ambulatory brain-computer interfaces: a pilot study with P300 signals,” in Proceedings of the International Conference on Advances in Computer Enterntainment Technology, 336–339. 10.1145/1690388.1690452

[B40] Lotte F. Guan C. (2011). Regularizing common spatial patterns to improve BCI designs: unified theory and new algorithms. IEEE Trans. Biomed. Eng. 58, 355–362. 10.1109/TBME.2010.2082539 20889426

[B41] Mason S. G. Birch G. E. (2003). A general framework for brain-computer interface design. IEEE Trans. Neural Syst. Rehabil. Eng. 11, 70–85. 10.1109/TNSRE.2003.810426 12797728

[B42] McCrea S. Geršak G. Novak D. (2016). Absolute and relative user perception of classification accuracy in an affective video game. Interact. Comput. 29, 271–286. 10.1093/iwc/iww026

[B43] Mihajlović V. Grundlehner B. Vullers R. Penders J. (2015). Wearable, wireless EEG solutions in daily life applications: what are we missing? IEEE J. Biomed. Health Inform. 19, 6–21. 10.1109/JBHI.2014.2328317 25486653

[B44] Minguillon J. Lopez-Gordo M. A. Pelayo F. (2017). Trends in EEG-BCI for daily-life: requirements for artifact removal. Biomed. Signal Process. Control 31, 407–418. 10.1016/j.bspc.2016.09.005

[B45] Mladenovic J. Mattout J. Lotte F. (2018). “A generic framework for adaptive EEG-based BCI training and operation,” in Brain-Computer Interfaces Handbook: Technological and Theoretical Advances, eds C. Nam, A. Nijholt, and F. Lotte (Boca Raton, FL: Taylor & Francis). 10.1201/9781351231954-31

[B46] Nam C. S. Nijholt A. Lotte F. (2018). Brain-Computer Interfaces Handbook: Technological and Theoretical Advances (Boca Raton, FL: CRC Press). 10.1201/9781351231954

[B47] Pfurtscheller G. Allison B. Z. Bauernfeind G. Brunner C. Solis Escalante T. Scherer R.. (2010). The hybrid BCI. Front. Neurosci. 4:3. 20582271 10.3389/fnpro.2010.00003 PMC2891647

[B48] Pillette L. (2019). Redefining and adapting feedback for mental-imagery based brain-computer interface user training to the learners' traits and states (Ph.D. thesis). Université de Bordeaux, Bordeaux, France. 10.3389/fnpro.2010.00003

[B49] Pope A. T. Bogart E. H. Bartolome D. S. (1995). Biocybernetic system evaluates indices of operator engagement in automated task. Biol. Psychol. 40, 187–195. 10.1016/0301-0511(95)05116-3 7647180

[B50] Rasmussen J. (1997). Risk management in a dynamic society: a modelling problem. Saf. Sci. 27, 183–213. 10.1016/S0925-7535(97)00052-0

[B51] Roy Y. Banville H. Albuquerque I. Gramfort A. Falk T. H. Faubert J. (2019). Deep learning-based electroencephalography analysis: a systematic review. J. Neural Eng. 16:051001. 10.1088/1741-2552/ab260c 31151119

[B52] Saha S. Baumert M. (2019). Intra-and inter-subject variability in EEG-based sensorimotor brain computer interface: a review. Front. Comput. Neurosci. 13:87. 10.3389/fncom.2019.00087 32038208 PMC6985367

[B53] Scerbo M. W. Freeman F. G. Mikulka P. J. (2003). A brain-based system for adaptive automation. Theor. Issues Ergon. Sci. 4, 200–219. 10.1080/1463922021000020891

[B54] Serim B. Jacucci G. (2019). “Explicating “implicit interaction”: An examination of the concept and challenges for research,” in Proceedings of the 2019 CHI Conference on Human Factors in Computing Systems, CHI'19 (New York, NY: Association for Computing Machinery), 1–16. 10.1145/3290605.3300647

[B55] Shenoy P. Krauledat M. Blankertz B. Rao R. Müller K.-R. (2006). Towards adaptive classification for BCI. J. Neural Eng. 3:R13. 10.1088/1741-2560/3/1/R02 16510936

[B56] Siemieniuch C. E. Sinclair M. A. (2006). Systems integration. Appl. Ergon. 37, 91–110. 10.1016/j.apergo.2005.06.012 16150416

[B57] Solovey E. Afergan D. Peck E. M. Hincks S. W. Jacob R. J. K. (2015). Designing implicit interfaces for physiological computing: guidelines and lessons learned using fNIRS. ACM Trans. Comput.-Hum. Interact. 21. 10.1145/2687926

[B58] Strait M. Scheutz M. (2014). What we can and cannot (yet) do with functional near infrared spectroscopy. Front. Neurosci. 8:117. 10.3389/fnins.2014.00117 24904261 PMC4033094

[B59] Sweeney K. T. Ward T. E. McLoone S. F. (2012). Artifact removal in physiological signals-practices and possibilities. IEEE Trans. Inform. Technol. Biomed. 16, 488–500. 10.1109/TITB.2012.2188536 22361665

[B60] Urigüen J. A. Garcia-Zapirain B. (2015). EEG artifact removal-state-of-the-art and guidelines. J. Neural Eng. 12:031001. 10.1088/1741-2560/12/3/031001 25834104

[B61] Vitorio R. Stuart S. Rochester L. Alcock L. Pantall A. (2017). fNIRS response during walking-artefact or cortical activity? A systematic review. Neurosci. Biobehav. Rev. 83, 160–172. 10.1016/j.neubiorev.2017.10.002 29017917

[B62] Wilson J. R. (2014). Fundamentals of systems ergonomics/human factors. Appl. Ergon. 45, 5–13. 10.1016/j.apergo.2013.03.021 23684119

[B63] Zander T. O. Kothe C. (2011). Towards passive brain-computer interfaces: applying brain-computer interface technology to human-machine systems in general. J. Neural Eng. 8:025005. 10.1088/1741-2560/8/2/025005 21436512

[B64] Zander T. O. Krol L. R. Birbaumer N. P. Gramann K. (2016). Neuroadaptive technology enables implicit cursor control based on medial prefrontal cortex activity. Proc. Natl. Acad. Sci. U.S.A. 113, 14898–14903. 10.1073/pnas.1605155114 27956633 PMC5206562

[B65] Zhang X. Yao L. Wang X. Monaghan J. McAlpine D. (2019). A survey on deep learning based brain computer interface: recent advances and new frontiers. arXiv. arXiv:1905.04149v5. 33171452

